# Roles of values in the risk factors of passive suicide ideation among young adults in the US and Japan

**DOI:** 10.3389/fpsyg.2023.1239103

**Published:** 2023-08-10

**Authors:** Kanako Taku, Hirokazu Arai

**Affiliations:** ^1^Oakland University, Rochester, MI, United States; ^2^Hosei University, Chiyoda, Japan

**Keywords:** value importance, value congruence, perceived burdensomeness, thwarted belongingness, depression

## Abstract

The present study examined how the importance of values and perceived value congruence with families, friends, and country would be associated with the risk factors of passive suicide ideation. Specifically, the study investigated the associations that the values and perceived congruence had with thwarted belongingness and perceived burdensomeness during the COVID-19 pandemic after controlling for the impact of depression levels. The data from the US and Japan demonstrated that the values such as cherishing family and friends and value congruence played a protective factor for Japanese participants; however, the associations differed among those in the US. Values such as enduring challenges played a protective factor for perceived burdensomeness in Japan whereas values such as cherishing family and friends played a protective factor and improving society was a risk factor for thwarted belongingness for those in the US. These results can be used to further understand the roles of values in mental health.

## Introduction

People sometimes realize what is most important in their lives when they experience life crises (Tedeschi et al., 2018). Some may realize they value relationships with family members, and others may realize they value their personal achievement, and yet others may value both equally without experiencing a conflict. One of the most comprehensive theories that provides systematic categorizations of personal values is Schwartz’ theory of basic human values (Schwartz, 2012). According to the theory, values refer to desirable goals that influence perceptions and emotions and motivate action. During the COVID-19 pandemic, for example in France, conservation values such as favoring stability, conformity, security, and preserving traditional practices, played an important role as a mediator between the perceived threat of the pandemic and compliance with social distancing and movement restrictions (Bonetto et al., 2021). Another study conducted in Spain illustrated a protective role of conservation values such as security and conformity directed people to adhere to self-protective behaviors such as social distancing during the pandemic (Tabernero et al., 2020). And yet, these studies also report that people hold various value contents, such as self-transcendence or hedonism, almost at the same level as conservation values, even though holding these values did not seem to significantly contribute to desirable actions, as values are beliefs (Schwartz, 2012) that people hold, whether or not they are practically useful or effective.

Self-identified importance in various value contents should be shaped by a combination of various personal and social factors, including culture. When some measures were applied to slow the speed of the pandemic, people from different cultures reacted differently. For example, in cultures where physical touch is not highly valued, people did not show strong reactions toward the government recommendation of no handshaking or hugging. In traditionally individualistic cultures such as the US, personal accomplishment may be more important than traditionally collectivistic cultures. Previous studies have demonstrated such cross-cultural differences, indicating values such as harmony and national security were valued more highly by Japanese, whereas comfortable life, wisdom, world peace, and freedom were valued more highly by Americans (Akiba and Klug, 1999).

Another aspect of values that should also be considered, beyond contents, is value congruence (Edwards and Cable, 2009). Previous studies have demonstrated the positive impact of value congruence between individuals and groups to which they belong, such as organizational and religious affiliations (Dunaetz et al., 2020). A meta-analytical study (Khaptsova and Schwartz, 2016) indicated that the positive effect of value congruence on life satisfaction held when controlling for various socio-demographic variables. Moreover, they found that the relationship between value congruence and life satisfaction was stronger when the independence of action value was less important. Since this meta-analytical study was conducted in one country (i.e., Russia), it is necessary to study how value congruence may play a role in mental health in other countries as well. One recent study (Elster and Gelfand, 2021) reported that the value-behavior relationship was stronger in “loose cultures (i.e., cultures that have weak social norms)” and was almost non-existent in “tight cultures (i.e., behavior being restricted by social constraints).” These findings suggest that people in tighter cultures such as Japan may not feel that they can choose to behave solely based on their personal values (i.e., weaker value-behavior relationship); thus, value congruence may serve a protective factor for their mental health. On the other hand, people in more loose cultures such as the US may feel that they can choose to behave based on their personal values; thus, value congruence may be of less importance.

In order to test these hypotheses, the current study focuses on two mental health factors that are proximate in suicidal ideation: thwarted belongingness and perceived burdensomeness (Van Orden et al., 2012). One pressing issue today is to identify a set of risk factors and protective factors for suicide. Suicide continues to be one of the major leading causes of death. Given its enormous psychological, societal, and economic impact, it is imperative to further study the mechanisms behind suicide. The current study focuses on two deprived interpersonal needs (i.e., thwarted belongingness and perceived burdensomeness) as the proximal causes of desire for suicide and examines how value contents as well as value congruence may serve as protective factors across two nations. A study conducted in Japan revealed that holding a value of importance in “cherishing friends and family” was associated with reduced suicidal ideation over a lifetime (Yasuma et al., 2019b), therefore similar associations were expected in the current study as well.

The current study has two purposes. First, it investigates how the perceived importance of various value contents (e.g., stable life, improving society) and perceived value congruence may vary across two nations – Japan and the US. Second, it tests if the importance of value contents would have a stronger impact than perceived value congruence on thwarted belongingness and perceived burdensomeness for people in the US, whereas the perceived congruence would show a stronger relationship for people in Japan.

## Method

### Participants and procedures

#### American sample

A total of 430 participants (18.1% male, 78.4% female, 2.6% non-binary or transgender, and 0.9% did not specify; mean age = 20.62, SD = 4.42) were recruited in the US between mid-May and mid-November of 2021. Participants were recruited through introductory psychology classes at a university in the Mid-western US. They received a course credit by participating in the questionnaire survey via Qualtrics. For the current study, the participants who passed the attention checks and typed their age were included to avoid potential bot responders. The final sample consisted of 393 (77.9% female) with a mean age of 20.55 (SD = 4.31). Sixty-five percent identified as White, 11.5% as Black or African American, 10.9% as Middle Eastern, 6.4% as Asian, and the remaining participants identified as other racial categories, such as American Indian.

#### Japanese sample

A total of 338 participants (49.4% male, 42.0% female, 0.9% non-binary or transgender, and 7.87% did not specify; mean age = 19.95, SD = 3.44) were recruited in various parts of Japan (36.1% from Kumamoto prefecture, 23.4% from Hokkaido prefecture, and the remaining participants were primarily from Hiroshima and Tokyo) between September and November of 2021. Participants were recruited using a snowball sampling method and participated in the same Qualtrics survey but with no incentives or course credits. The same criteria were applied to determine the final sample. The final sample consisted of 255 (47.1% female) with a mean age of 20.03 (SD = 3.69). All but one identified themselves as Asian (one responded “prefer not to answer”). The study was approved by the university institutional review board.

### Measures

#### Importance of value contents

The degree to which each value is important or unimportant was measured using the shortened and adapted version of the Portrait Values Questionnaire. The “Recently Revised Portrait Value Questionnaire” (PVQ-RR: Schwartz and Cieciuch, 2022) consists of 57 items based on the Schwarz theory of basic values. For the current study, we used 11 items that had been adapted in previous studies (Yasuma et al., 2019a,b). These 11 items (e.g., “avoiding causing trouble,” “financial success,” etc.) were presented with an instruction, “how important are the following values in your life?” Participants responded using a 7-point Likert scale, ranging from 1 (extremely unimportant) to 7 (extremely important). Cronbach’s alpha coefficients for these 11 items were 0.86 for the current US sample and 0.84 for the current Japanese sample. However, because these 11 items were not intended to comprehensively capture the Schwartz basic value categories, each item was used in the current study without aggregating them into higher-order value dimensions.

#### Perceived value congruence

The levels of perceived congruence in values were measured using the following questions that were developed for the current study, “Please select the answer that most describes the consistency of your values in relation to…” The first was “your values and your family’s values,” the second was “your values and your friends’ values,” and the third was “your values and your country’s values.” For each of these pairs, participants used a 7-point scale ranging from 1 (*extremely inconsistent*) to 7 (*perfectly consistent*). Cronbach’s alpha coefficients for these 3 items were 0.58 for the current US sample and 0.77 for the current Japanese sample. However, because these 3 items were intended to capture the perceived congruence with family, friends, and country, respectively, each item was used in the current study without aggregating them into higher-order value congruence.

#### Depression

Depression was assessed using a 7-item depression subscale of the Hospital Anxiety and Depression Scale (Zigmond and Snaith, 1983; Higashi et al., 1996). Participants responded to these items (e.g., “I feel as if I am slowed down”) using a 4-point scale (0 = never, 1 = sometimes, 2 = often, and 3 = always). Cronbach’s alpha coefficients were 0.79 for the current US sample and 0.69 for the current Japanese sample.

#### Thwarted belongingness and perceived burdensomeness

Two of the proximal causes of desire for suicide, thwarted belongingness and perceived burdensomeness, were measured using the Interpersonal Needs Questionnaire (INQ: Van Orden et al., 2012; Aiba et al., 2019). Participants responded using a 7-point scale from 1 (*not at all true for me*) to 7 (*very true for me*). The 9-item subscale of thwarted belongingness (e.g., “These days, I feel disconnected from other people”) showed a satisfactory internal consistency (Cronbach’s alpha coefficients were 0.88 for the US sample and 0.86 for the Japanese sample). The 6-item subscale of perceived burdensomeness (e.g., “These days I think I am a burden on society”) also showed a good internal consistency (alpha was 0.94 for the US sample and 0.94 for the Japanese sample).

### Data analyses

First, two mixed ANOVA were conducted to examine the cross-national differences in the levels of importance of 11 value contents and three aspects of perceived value congruence. Second, a hierarchical regression analysis was conducted to test if value contents or value congruence would be more strongly associated with thwarted belongingness and perceived burdensomeness. For each country separately, demographics as well as depression levels were first entered into the model. Importance of the 11 values was then entered in the second step. Finally, the three aspects of perceived value congruence between themselves and their family, friends, and country were added. As recommended in Schwartz (2012), centering value scores have been commonly used in measures assessing personal values; thus, Z scores were used for the regression analyses. All analyses were conducted using IBM SPSS Statistics (Version 27; IBM 2021).

## Results

### Cross-national and within-national differences in the importance of value contents

A mixed ANOVA with nation as a between-factor and the importance of 11 value contents as within-factors showed a significant main effect of nation, *F* (1, 622) = 27.36, *p* < 0.001, and values, *F* (10, 613) = 99.81, *p* < 0.001. The interaction effects were also significant, *F* (10, 613) = 42.86, *p* < 0.001, partial eta squared = 0.41. As shown in Table 1, follow up analyses revealed that values such as positive evaluation, financial success, improving society, graduating from school and stable lifestyle were perceived to be more important for the US participants than the Japanese participants, whereas enduring challenges was valued more highly for the Japanese participants. In both countries, cherishing family and friends was valued highly and social influence was least valued.

**Table 1:** Cross-national comparisons in the importance of value contents.

	US				Japan				*p*
	*M*	95% CI	SE		*M*	95% CI	SE		
1. Avoiding causing trouble	5.84	[5.71, 5.96]	0.06	b	5.73	[5.58, 5.88]	0.08	b	
2. Positive evaluation	5.91	[5.80, 6.02]	0.05	b	5.67	[5.54, 5.80]	0.07	b	^**^
3. Belief	5.76	[5.64, 5.88]	0.06	c	5.88	[5.73, 6.03]	0.08	b	
4. Financial success	6.05	[5.95, 6.16]	0.05	b	5.58	[5.45, 5.71]	0.07	c	^**^
5. Improving society	5.75	[5.63, 5.88]	0.06	c	5.34	[5.18, 5.49]	0.08	c	^**^
6. Interest	5.94	[5.85, 6.03]	0.05	b	6.02	[5.90, 6.13]	0.06	b	
7. Social influence	4.77	[4.62, 4.92]	0.08	e	4.58	[4.39, 4.76]	0.10	d	
8. Enduring challenging	5.36	[5.23, 5.49]	0.07	d	5.87	[5.71, 6.04]	0.08	b	^**^
9. Cherishing family and friends	6.39	[6.30, 6.47]	0.04	a	6.48	[6.37, 6.59]	0.06	a	
10. Graduating from school	6.45	[6.32, 6.58]	0.07	a	4.48	[4.32, 4.64]	0.08	d	^**^
11. Stable lifestyle	6.55	[6.46, 6.64]	0.05	a	5.91	[5.80, 6.03]	0.06	b	^**^

^**^p < 0.01.

Different letters in each country showed significant within-culture differences at *p* < 0.01.

### Cross-national and within-national differences in value congruence

A mixed ANOVA revealed a significant main effect of nation, *F* (1, 634) = 12.49, *p* < 0.001, and value congruence, *F* (2, 633) = 113.10, *p* < 0.001. Interaction effects were also significant, *F* (2, 633) = 63.94, *p* < 0.001, partial eta squared = 0.17. As shown in Table 2, follow up analyses showed that the congruence was greater with family’s and friends’ values among the US participants, whereas the congruence with country’s values was greater in the Japanese counterpart. Within the US, consistency was higher with friends than with family, and the congruence with country’s values was the least. For the Japanese participants, overall consistency was rather moderate; thus, the only difference that was detected was value congruence with friends and with country (i.e., the congruence with country’s values was lower; however, it was still higher than the US counterpart).

**Table 2:** Cross-national comparisons in the value congruence.

	US				Japan				*p*
	*M*	95% CI	SE		*M*	95% CI	SE		
Self and family’s values	5.20	[5.05, 5.35]	0.08	b	4.59	[4.40, 4.78]	0.10		^**^
Self and friends’ values	5.47	[5.35, 5.59]	0.06	a	4.62	[4.46, 4.77]	0.08	a	^**^
Self and country’s values	3.88	[3.73, 4.03]	0.08	c	4.39	[4.20, 4.58]	0.10	b	^**^

^**^p < 0.01.

Different letters in each country showed significant within-country differences at *p* < 0.01.

### Association between importance of values, value congruence, and suicidal ideation

First hierarchical regression analysis was conducted with perceived burdensomeness. For the US sample, the overall model for Step 1 was significant, *F* (3, 349) = 37.19, *p* < 0.001, adjusted *R*^2^ = 0.24. Depression was the only significant predictor, *b* = 0.67, 95% CI [0.54, 0.80], SE = 0.06, β = 0.49, *p* < 0.001. The model was improved at Step 2, *F* (14, 338) = 9.77, *p* < 0.001, adjusted *R*^2^ = 0.26, Δ*R*^2^ = 0.05. In addition to the positive associations that depression had with perceived burdensomeness, one value, “enduring active challenges,” was a negative predictor, serving as a protective factor, b = −0.25, 95% CI [−0.39, −0.10], SE = 0.08, β = −0.18, *p* = 0.001. At Step 3, the model was not significantly improved, *F* (17, 335) = 8.47, *p* < 0.001, adjusted *R*^2^ = 0.27, Δ*R*^2^ = 0.01, indicating that value congruence was not associated with perceived burdensomeness (Table 3). For the Japanese sample, the overall model for Step 1 was significant, *F* (3, 212) = 18.41, *p* < 0.001, adjusted *R*^2^ = 0.20. Depression was the only significant predictor, *b* = 0.67, 95% CI [0.49, 0.84], SE = 0.09, β = 0.67, *p* < 0.001. The model was significantly improved at Step 2, *F* (14, 201) = 6.78, *p* < 0.001, adjusted *R*^2^ = 0.27, Δ*R*^2^ = 0.11. In addition to the positive associations that depression had with perceived burdensomeness, one value, “cherishing family and friends,” was a negative predictor, serving as a protective factor, b = −0.43, 95% CI [−0.66, −0.20], SE = 0.12, β = −0.25, *p* < 0.001. Finally, at Step 3, the model was again significantly improved, *F* (17, 198) = 7.17, *p* < 0.001, adjusted *R*^2^ = 0.33, Δ*R*^2^ = 0.06, indicating that value congruence with friends served a potentially protective factor, b = −0.22, 95% CI [−0.42, −0.02], SE = 0.10, β = −0.22, *p* = 0.03 (Table 3).

**Table 3:** Regression models predicting perceived burdensomeness at Step 3.

	US				Japan			
	b	CI	SE	β	b	CI	SE	β
Gender	−0.09	[−0.42, 0.23]	0.17	−0.03	0.35	[0.01, 0.70]	0.17	0.12^*^
Age	−0.04	[−0.15, 0.07]	0.06	−0.03	−0.12	[−0.30, 0.06]	0.09	−0.08
Depression	0.57	[0.44, 0.71]	0.07	0.42^**^	0.38	[0.19, 0.56]	0.09	0.26^**^
1. Avoiding causing trouble	0.10	[−0.06, 0.25]	0.08	0.07	0.11	[−0.05, 0.28]	0.08	0.09
2. Positive evaluation	−0.06	[−0.23, 0.12]	0.09	−0.04	0.04	[−0.15, 0.23]	0.10	0.03
3. Belief	−0.04	[−0.19, 0.11]	0.07	−0.03	−0.25	[−0.49, −0.01]	0.12	−0.16^*^
4. Financial success	−0.01	[−0.20, 0.18]	0.10	−0.01	−0.17	[−0.38, 0.04]	0.11	−0.12
5. Improving society	0.16	[−0.03, 0.34]	0.09	0.10	0.18	[−0.01, 0.37]	0.10	0.15
6. Interest	−0.07	[−0.24, 0.10]	0.09	−0.05	−0.29	[−0.53, −0.05]	0.12	−0.20^*^
7. Social influence	0.15	[−0.00, 0.29]	0.08	0.11	0.08	[−0.14, 0.30]	0.11	0.06
8. Enduring challenging	−0.23	[−0.38, −0.08]	0.08	−0.17^**^	0.07	[−0.19, 0.34]	0.13	0.05
9. Cherishing family and friends	−0.11	[−0.29, 0.06]	0.09	−0.08	−0.36	[−0.60, −0.12]	0.12	−0.21^**^
10. Graduating from school	0.14	[−0.15, 0.42]	0.15	0.06	0.00	[−0.19, 0.19]	0.10	0.00
11. Stable lifestyle	−0.02	[−0.27, 0.24]	0.13	−0.01	0.19	[−0.02, 0.40]	0.11	0.14
Value congruence - family	−0.14	[−0.29, 0.01]	0.08	−0.10	−0.11	[−0.32, 0.09]	0.10	−0.08
Value congruence - friends	−0.02	[−0.18, 0.14]	0.08	−0.01	−0.22	[−0.41, −0.02]	0.10	−0.16^*^
Value congruence - country	−0.05	[−0.18, 0.08]	0.07	−0.04	−0.19	[−0.43, 0.05]	0.12	−0.11

^*^p < 0.05, ^**^p < 0.01.

Second hierarchical regression analysis was conducted with thwarted belongingness. For the US sample, the model for Step 1 was significant, *F* (3, 349) = 64.84, *p* < 0.001, adjusted *R*^2^ = 0.35. Depression was the only significant predictor, *b* = 0.77, 95% CI [0.66, 0.88], SE = 0.06, β = 0.60, *p* < 0.001. The model was improved at Step 2, *F* (14, 338) = 20.11, *p* < 0.001, adjusted *R*^2^ = 0.43, Δ*R*^2^ = 0.10. In addition to the positive associations that depression had with thwarted belongingness, one value, “improving society,” had a positive association, serving as a risk factor, whereas “cherishing family and friends” was a negative predictor, serving as a protective factor. Finally, at Step 3, the model was significantly improved, *F* (17, 335) = 17.82, *p* < 0.001, adjusted *R*^2^ = 0.45, Δ*R*^2^ = 0.02, indicating that value congruence with family was associated with thwarted belongingness (Table 4). For the Japanese sample, the model for Step 1 was significant, *F* (3, 212) = 25.24, *p* < 0.001, adjusted *R*^2^ = 0.25. Depression was the only significant predictor. The model was improved at Step 2, *F* (14, 201) = 8.69, *p* < 0.001, adjusted *R*^2^ = 0.33, Δ*R*^2^ = 0.11. In addition to the positive associations that depression had with thwarted belongingness, age served a protective factor, b = −0.19, 95% CI [−0.33, −0.06], SE = 0.07, β = −0.17, *p* < 0.01, so as one value, “cherishing family and friends,” b = −0.25, 95% CI [−0.42, −0.09], SE = 0.08, β = −0.20, *p* < 0.01. Finally, at Step 3, the model was significantly improved, *F* (17, 198) = 8.21, *p* < 0.001, adjusted *R*^2^ = 0.36, Δ*R*^2^ = 0.04, indicating that value congruence with friends served a protective factor, b = −0.19, 95% CI [−0.34, −0.05], SE = 0.07, β = −0.19, *p* < 0.01 (Table 4).

**Table 4:** Regression models predicting thwarted belongingness at Step 3.

	US				Japan			
	b	CI	SE	β	b	CI	SE	β
Gender	0.28	[0.01, 0.54]	0.14	0.09^*^	0.06	[−0.19, 0.31]	0.13	0.06
Age	0.08	[−0.01, 0.17]	0.05	0.07	−0.17	[−0.30, −0.04]	0.07	−0.17^*^
Depression	0.61	[0.50, 0.72]	0.06	0.47^**^	0.34	[0.21, 0.48]	0.07	0.34^**^
1. Avoiding causing trouble	0.11	[−0.02, 0.23]	0.06	0.08	0.01	[−0.10, 0.13]	0.06	0.01
2. Positive evaluation	−0.16	[−0.30, −0.01]	0.07	−0.11^*^	0.07	[−0.07, 0.21]	0.07	0.07
3. Belief	−0.02	[−0.13, 0.10]	0.06	−0.01	−0.06	[−0.23, 0.12]	0.09	−0.05
4. Financial success	−0.05	[−0.20, 0.10]	0.08	−0.03	−0.01	[−0.16, 0.14]	0.08	−0.01
5. Improving society	0.20	[0.05, 0.35]	0.08	0.14^**^	−0.01	[−0.15, 0.13]	0.07	−0.01
6. Interest	−0.12	[−0.26, 0.03]	0.07	−0.09	−0.09	[−0.27, 0.08]	0.09	−0.09
7. Social influence	−0.01	[−0.13, 0.12]	0.06	−0.00	−0.00	[−0.16, 0.16]	0.08	−0.00
8. Enduring challenging	−0.00	[−0.12, 0.12]	0.06	−0.00	−0.17	[−0.36, 0.02]	0.10	−0.15
9. Cherishing family and friends	−0.26	[−0.40, −0.12]	0.07	−0.19^**^	−0.21	[−0.38, −0.04]	0.09	−0.16^*^
10. Graduating from school	−0.23	[−0.46, 0.00]	0.12	−0.10	0.16	[0.02, 0.30]	0.07	0.16^*^
11. Stable lifestyle	0.13	[−0.08, 0.33]	0.11	0.08	−0.00	[−0.15, 0.15]	0.08	−0.00
Value congruence - family	−0.13	[−0.26, −0.01]	0.06	−0.10^*^	−0.05	[−0.20, 0.10]	0.08	−0.05
Value congruence - friends	−0.12	[−0.24, 0.01]	0.07	−0.08	−0.19	[−0.34, −0.05]	0.07	−0.19^**^
Value congruence - country	−0.03	[−0.14, 0.07]	0.05	−0.03	−0.02	[−0.19, 0.16]	0.09	−0.01

^*^p < 0.05, ^**^p < 0.01.

## Discussion

The first aim of the current study was to examine cross-national and within-national individual differences in the importance of value contents and value congruence. Results indicated that only one value “enduring challenges” was perceived more importantly for participants in Japan than those in the US and many other values such as positive evaluation, financial success, improving society, graduating from school, and stable lifestyle, were perceived more importantly for those in the US. Because values are ordered by importance, and the relative importance of multiple values guides action, researchers assessing contents of values are often recommended not to use absolute scores Schwartz, 2012. This is particularly important if the researchers aim to test theory-driven dimensionality of basic human values (Ponikiewska et al., 2020) from a cross-cultural perspective (Cieciuch et al., 2014). However, the absolute levels of ratings that the current study used seem very informative. For example, 67.1% of the US sample responded “stable lifestyle” as extremely important by choosing “7” out of 1–7 scale compared to only 32.1% of the Japanese sample. Because values that are placed at higher priority are likely to guide one’s attitudes and actions, whether the highest importance was given or not may not be of importance, especially given that the majority of values were placed lower by people in Japan than those in the US. And yet, the fact that none of the values was perceived “extremely important” for a certain percentage of people, especially in Japan, may challenge a value theory in general, because the theory assumes everyone should have something important values. The current results raise a question: if more and more people feel that “nothing seems important to me,” then how will that guide their behaviors? The argument for relative importance may need a threshold because if the perceived importance is too mild, if not weak, none of the values may become activated.

The second aim of the current study was to investigate the associations that the importance of value contents and perceived value congruence had with thwarted belongingness and perceived burdensomeness. The hypotheses were supported for people in Japan but only partially supported for people in the US. Regarding the Japanese sample, a robust protective factor for perceived burdensomeness and thwarted belongingness was “cherishing family and friends,” consistent with a previous study (Yasuma et al., 2019b). This value is suggested to be classified as a benevolence value based on Schwartz’s theory of basic values, which has been shown to be anxiety-free with social focus (Schwartz and Cieciuch, 2022). Furthermore, value congruence with friends also served as a protective factor. This result is consistent with a study conducted in Israel (Benish-Weisman et al., 2020), another culture that is traditionally collectivistic. Because of the collectivistic characteristics, coupled with a homogeneous society, shared values are likely to play a critical role in mental health for people in Japan, and the current results support that, which is also consistent with the cultural fear of exclusion (Wilberg, 2012).

Regarding the US sample, patterns were different between the two deprived interpersonal needs. For perceived burdensomeness, placing a value on enduring challenges served a protective factor. This type of value is related to openness to change, which is also a part of grit and resiliency that are known to moderate the relationships between trauma and suicidal ideation (Marie et al., 2019). However, this value was not associated with thwarted belongingness. Instead, just like the participants in Japan, the value of cherishing family and friends served as a protective factor. Interestingly, however, the value of improving society served as a risk factor for thwarted belongingness. We can speculate that one’s perceptions of needs for improvement in their own community might come from their observations or beliefs that others do not recognize the needs, which in turn may elicit a sense of frustration or isolation; thus, thwarted belongingness. The potentially important role of value congruence over and above the importance of value contents was expected to only be found among people in Japan given the diverse society of the US. However, although weak, value congruence with family served as a protective factor for thwarted belongingness for people in the US, as opposed to value congruence with friends found in Japan. The current study is unable to answer why this was the case. Future studies should address how value congruence plays a role in a sense of isolation and suicidal ideation.

Another future direction may aim to reveal the mechanisms behind the changes in values. The malleability of values has been suggested. Bonetto et al. (2021), for example, illustrated some forms of values such as achievement were placed lower whereas other values such as conformity and security were placed higher during the pandemic than usual. Given that values guide attitudes and actions and seem to affect the perceptions of deprived interpersonal needs, it will be critical to study under what conditions changes may occur in the relative importance of values within an individual and culture. One study suggested that changes in values, or at least a temporary adjustment of basic human values, in order to adapt to the COVID-19 pandemic may be more pronounced among individuals that are more sensitive to situational dynamics (i.e., people with low emotional stability) (Fischer et al., 2021). If so, various positive changes in values that were reported by traumatized people, known as post-traumatic growth (Tedeschi et al., 2018), might have been affected by the individuals’ sensitivities. This speculation should be tested in future research.

In the interpretation of our results, there are some limitations. First, this study adapted the 11 value contents that were selected in previous studies (Yasuma et al., 2019a). Although the researchers did not provide the rationale behind their selection, these 11 items may not fully cover the full range of values that have been universally identified. For example, Schwartz’ theory consists of four higher order values that form two dimensions. One is self-transcendence - self-enhancement dimension, and the other is openness to change - conservation dimension. The 11 items that the current study adopted failed to capture the dimension of self-transcendence, which could play an important role in perceived burdensomeness, because one study demonstrated that activating self-transcendence values promoted prosocial behaviors during the COVID-19 pandemic (Russo et al., 2022). Future research should use a full scale of basic values so that each value is structured as a circular continuum hierarchical model. Similarly, value congruence was assessed using self-report methodologies, that is, conscious level of “perceived” discrepancies. In order to evaluate how objective value congruence may affect mental health, it is necessary to calculate the discrepancies between an individual’s importance of values and the groups’ aggregated importance of values; however, such method also may have its disadvantages, knowing that individuals belong to multiple groups and communities. The second limitation is related to sampling. An unbalanced gender ratio with limited sample size must have affected our results. We also did not collect socio economic status of the participants. Sampling equivalence is difficult to meet for any cross-cultural studies. But the cross-national differences that were identified in the current study might have been due to the cross-religious differences, given that more Christians were included in the US sample and more non-religious individuals were included in Japan. This is important, because values are often closely tied to religious faith (Saroglou et al., 2004) and non-monotheistic religions and the nature of causality between values and religiosity has not been fully elucidated (Sagiv and Schwartz, 2022). The relatively low appreciation of value among the Japanese may be related to the large number of non-religious individuals. Similarly, the data we presented here were collected during the COVID-19 pandemic, but because it was a cross-sectional research design and we were not able to compare how the findings might differ from pre-COVID or post-COVID, we could not know how the results we obtained were due to the COVID-19 pandemic. It is critical to replicate the current study.

In summary, we investigated the cross-national and individual differences within each nation in the importance of values and value congruence during the COVID-19 pandemic. We also demonstrated the associations that these two aspects of values had with thwarted belongingness and perceived burdensomeness. Our data from two countries, albeit with some limitations, shed light on the multiple paths for future studies that may further refine the Interpersonal Theory of Suicide (Chu et al., 2017, for a review; Van Orden et al., 2005). When it comes to the moment of actions, personal importance of values and perceived value congruence may be the factors that play a key role.

## Data availability statement

The raw data supporting the conclusions of this article will be made available by the authors, without undue reservation.

## Ethics statement

The studies involving human participants were reviewed and approved by Institutional Review Board of Oakland University. The patients/participants provided their written informed consent to participate in this study.

## Author contributions

KT and HA involved in conception and design of study and interpreted the data. KT collected data and analyzed the data and drafted the first manuscript. HA revised the manuscript critically and made important intellectual contribution. All authors contributed to the article and approved the submitted version.

## Funding

This work was supported by JSPS KAKENHI Grant Numbers 22 K11481.

## Conflict of interest

The authors declare that the research was conducted in the absence of any commercial or financial relationships that could be construed as a potential conflict of interest.

## Publisher’s note

All claims expressed in this article are solely those of the authors and do not necessarily represent those of their affiliated organizations, or those of the publisher, the editors and the reviewers. Any product that may be evaluated in this article, or claim that may be made by its manufacturer, is not guaranteed or endorsed by the publisher.
